# Applications of Multiparameter Flow Cytometry in the Diagnosis, Prognosis, and Monitoring of Multiple Myeloma Patients

**DOI:** 10.3390/diseases13100320

**Published:** 2025-10-01

**Authors:** Dimitrios Leonardos, Leonidas Benetatos, Elisavet Apostolidou, Epameinondas Koumpis, Lefkothea Dova, Eleni Kapsali, Ioannis Kotsianidis, Eleftheria Hatzimichael

**Affiliations:** 1 Department of Hematology, Faculty of Medicine, School of Health Sciences, University of Ioannina, 45500 Ioannina, Greece; d.leonardos@uoi.gr (D.L.); lbenet@uoi.gr (L.B.); e.apostolidou@uoi.gr (E.A.); an.koumpis@uoi.gr (E.K.); ekapsali@uoi.gr (E.K.); 2 Molecular Biology Laboratory, University Hospital of Ioannina, Niarchos Ave, 45100 Ioannina, Greece; ldova@uoi.gr; 3 Department of Hematology, University Hospital of Alexandroupolis, Democritus University of Thrace, 68100 Alexandroupolis, Greece; ikotsian@med.duth.gr

**Keywords:** multiple myeloma, flow cytometry, measurable residual disease, immunophenotype, circulating plasma cells

## Abstract

Multiple myeloma (MM) is one of the most common hematological malignancies and remains incurable. However, the survival of multiple myeloma patients has significantly increased due to the implementation of novel therapies along with autologous stem cell transplantation, changing the natural history of the disease. Consequently, there is an unmet need for more sensitive response assessment techniques capable of quantifying minimal tumor burden to identify patients at higher risk of early relapse. Multiparameter flow cytometry (MFC) is an essential tool for diagnosing and monitoring patients with various hematological conditions and has recently gained prominence in identifying, characterizing, and monitoring malignant plasma cells. The implementation of Next-Generation Flow (NGF) by EuroFlow aims to overcome the pitfalls of conventional MFC, including lack of standardization and lower sensitivity, by offering standardized and optimized protocols for evaluating response depth. Both MFC and NGF have wide-ranging applications in MM for diagnosis and measurable residual disease (MRD) monitoring. Plasma cell identification and clonality evaluation through MFC and NGF assist in diagnostic workup and are routinely used to assess therapeutic response through MRD analysis. Additionally, flow cytometry is applied for circulating tumor plasma cell (CTPC) enumeration, which has demonstrated significant prognostic value. Immune composition studies through MFC may provide better understanding of disease biology. Furthermore, MFC provides additional information about other bone marrow cell populations, assessing cellularity, immunophenotypic characteristics of plasma cells, and possible hemodilution. This review explores the applications of MFC and NGF in MM, highlighting their roles in diagnosis, response assessment, and prognosis. Beyond their established use in MRD monitoring, flow cytometry-derived immunophenotypic profiles show strong potential as cost-effective prognostic tools. We advocate for future studies to validate and integrate these markers into risk stratification models, complementing cytogenetic analyses and guiding individualized treatment strategies.

## 1. Introduction

Multiple myeloma (MM) is a malignancy of terminally differentiated plasma cells in the bone marrow. These malignant plasma cells produce a monoclonal immunoglobulin (paraprotein), that can be detected in serum or urine, contributing to the disease’s pathophysiology. The accumulation of these clonal plasma cells leads to destruction of the bone marrow microenvironment, impairing normal hematopoiesis and suppressing polyclonal immunoglobulin production [1].

With an estimated 6 new cases per 100,000 people annually, MM is one of the most common hematological malignancies, second to Non-Hodgkin lymphomas, accounting for 10% of all hematological diseases and 1% of all neoplasms in general [1,2]. Monoclonal gammopathy of undetermined significance (MGUS) is usually a preceding stage, with a progression risk of 1% per year in the first 5 years [3]. In total, approximately 15% of MGUS patients will advance to symptomatic MM at some time during their disease course [4].

Recent advances in understanding MM biology, improving diagnostic techniques, and developing novel therapeutic approaches have revolutionized patient management. Enhanced diagnostic criteria, the use of highly sensitive monitoring tools, and integration of novel therapeutic agents have all contributed to significant progress in MM outcomes [2,5]. The use of three- or four-drug combinations, in conjunction with autologous stem cell transplantation and CAR-T cell therapy has resulted in deeper responses and improved outcomes [5,6]. Implementation of such regimens achieves overall response rates of nearly 100%, with a high percentage of near-complete remissions [7,8]. Subsequently, overall survival (OS) has been significantly prolonged in recent years.

Despite these very promising results, multiple myeloma patients will eventually relapse [5,9,10]. This situation highlights the limitations of conventional response assessment and monitoring (serum electrophoresis, immunofixation, quantification of serum free light chains) and emphasizes the need for more sensitive response assessment techniques capable of quantifying minute quantities of cancer cells, providing greater insight into response depth and improving long-term prognostication [4,8,11]. Currently, response assessment and measurable residual disease (MRD) quantification constitute one of the most compelling research fields in MM, as they enable better prognostication and, hopefully, clinical decision-making.

**Figure 1:**
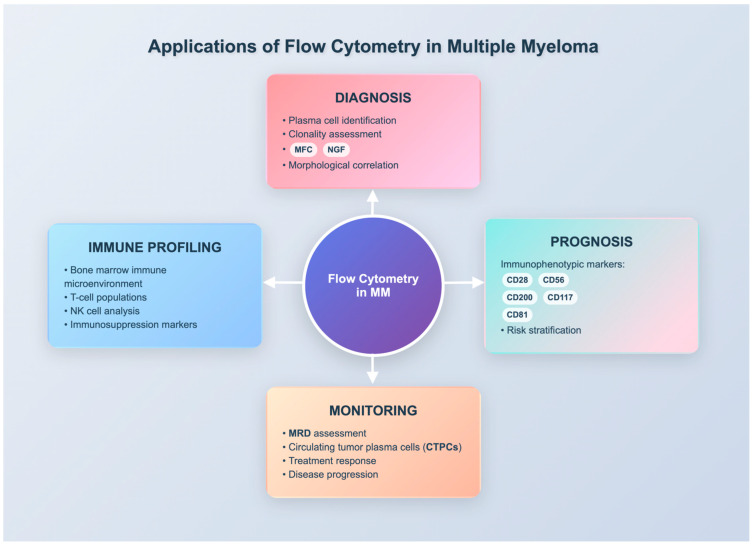
Applications of flow cytometry in multiple myeloma, including diagnosis, prognosis, MRD monitoring, and immune profiling. Abbreviations: MFC = multiparameter flow cytometry; NGF = next generation flow; MRD = measurable residual disease; CTPCs = circulating tumor plasma cells; NK = natural killer.

Multiparameter flow cytometry (MFC) has become an essential tool across several disciplines, including immunology, virology, hematology, oncology, and infectious diseases. In hematology, it is predominantly used for diagnosing and monitoring various hematological diseases. Multiparameter flow cytometry (MFC) has long been used in the diagnostic and prognostic evaluation of hematological malignancies, including multiple myeloma. It allows rapid and cost-effective immunophenotypic characterization of plasma cells, with wide clinical availability [12]. However, conventional MFC is limited by variable antibody panels, lack of standardization, and a sensitivity typically restricted to 10^−4^–10^−5^, which may underestimate measurable residual disease (MRD) [13,14]. Next Generation Flow (NGF) has addressed some of these issues by providing standardized EuroFlow panels and higher sensitivity (10^−6^), but implementation remains limited outside specialized centers. Next-generation sequencing (NGS) offers similar or even greater sensitivity but requires baseline samples, is more costly, and is less widely accessible [15]. Thus, while multiple technologies are available for MRD assessment, challenges such as inter-laboratory variability, accessibility, and standardization underscore the ongoing need to optimize and harmonize flow cytometry approaches in MM [13]. MFC was first explored in monoclonal gammopathy research in the 1980s [12,15]. In 2002, two groups first demonstrated the clinical use of flow cytometry for assessing residual disease and therapeutic response in MM patients [15,16,17]. Recent advancements, including the use of additional fluorochromes and optimized protocols, have increased the sensitivity of flow cytometry to approximately 10^−6^, making it comparable to molecular assays. Further gains toward 10^−7^–10^−8^ appear technically achievable; however, their routine feasibility in diagnostic laboratories remains uncertain due to sample and resource constraints. While such ultra-sensitive detection could further refine prognostic discrimination, its true clinical value and practicality in everyday practice remain to be determined [18]. The major clinical applications of flow cytometry in multiple myeloma, including diagnosis, prognosis, MRD monitoring, and immune profiling are summarized in Figure 1.

## 2. Advantages and Disadvantages of Multiparameter Flow Cytometry

Multiparameter flow cytometry (MFC) has emerged as an essential tool for the quantification and characterization of plasma cells in different biological samples. It addresses several key clinical needs, including: (i) rapid and accurate diagnostic work-up by distinguishing clonal from polyclonal plasma cells; (ii) measurable residual disease (MRD) monitoring for response assessment; and (iii) risk stratification through the identification of aberrant immunophenotypic profiles [10,13]. By providing cost-effective and timely results, MFC helps overcome challenges in establishing clonality, evaluating treatment efficacy, and identifying patients at higher risk of progression. Nevertheless, as discussed below, the method is subject to important limitations in sensitivity, reproducibility, and inter-laboratory standardization [10]. Response assessment and monitoring in MM patients requires techniques that meet several important criteria: high sensitivity, broad applicability, standardization, and cost-effectiveness [10,19,20]. Flow cytometry-based methods offer a balance between accuracy, availability, and cost, making them highly attractive for monitoring. Conventional flow cytometry is applicable to virtually all MM patients (>95%), and its wide availability and reproducibility facilitate routine use. These appealing features of MFC facilitate diagnostic workup in daily clinical practice by providing accurate diagnosis with low turnaround time and cost-effectiveness [19,21,22].

The sensitivity of conventional 4–6 color flow cytometry is approximately 10^−4^, which is lower than that of next-generation sequencing (NGS). A notable advantage of flow cytometry in patient monitoring is that a diagnostic baseline sample is not required, allowing MRD status assessment at any point during therapy [13]. Moreover, MFC provides simultaneous detection of both intracellular and extracellular antigens and quantitative evaluation of various bone marrow cell populations based on their immunophenotypic antigen expression [13,14,23]. Additionally, MFC may provide intra-sample quality assessment for possible hemodilution, a pitfall that may result in underestimation of neoplastic tumor burden, by identifying other cellular populations [24].

Despite its strengths, flow cytometry has several limitations that have prompted further research and optimization. MFC applied to bone marrow samples has limited value in predicting patchy or extramedullary disease, as bone marrow aspirates may not reflect overall tumor burden [18,22]. Moreover, fresh sample collection and analysis, preferably within 24–36 h, is necessary for cell viability and immunophenotypic integrity preservation. Four- to six-color flow cytometers have limited sensitivity compared to NGS and other molecular methods in assessing MRD, highlighting the need for upgraded flow cytometry assays [18,24].

Furthermore, the lack of standardization and consensus in flow cytometry, including variability in antibody panels, numbers of acquired cells, and MRD positivity criteria, contributes to inconsistent results across different laboratories [21,22,24]. Inter-laboratory variability in sample preparation, antibody panel construction, gating strategies, and data interpretation has limited the comparability of MRD results. Ongoing international efforts, including updated consensus recommendations on MRD assessment (IMWG [4], EMN [25] and harmonization initiatives such as standardized data analysis pipelines [24], are essential to ensure that MRD results generated across different centers are reproducible, comparable, and clinically actionable. A laboratory survey conducted by Flanders et al. demonstrated considerable variability between laboratories, resulting in up to 100-fold differences in assay sensitivity. The survey revealed substantial discrepancies regarding antibody panel composition, sample staining, data acquisition, result reporting, and quality requirements. Thus, it remains clear that implementing a reference method is mandatory to provide uniformity in analysis and reporting across laboratories [24,26]. Principal differences in MRD assessment techniques are presented in Table 1.

**Figure 2:**
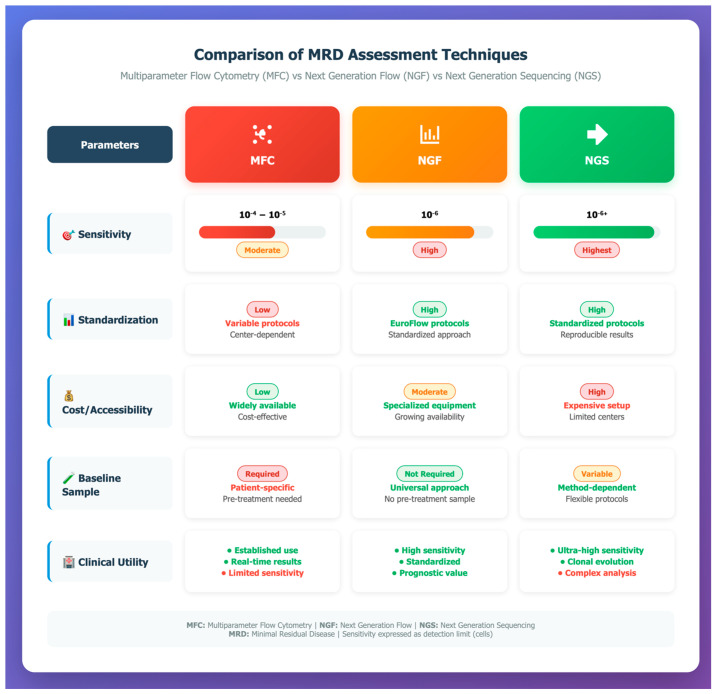
Comparison of MRD assessment techniques in multiple myeloma: MFC, NGF, and NGS across sensitivity, standardization, accessibility, and clinical utility. Abbreviations: MFC = multiparameter flow cytometry; NGF = next generation flow; NGS = next generation sequencing; MRD = measurable residual disease.

A visual comparison of MFC, NGF, and NGS for MRD assessment is presented in Figure 2, complementing the data shown in Table 1.

## 3. Next Generation Flow (NGF)

In response to these challenges, the EuroFlow Consortium introduced NGF in 2017 as a standardized and highly sensitive method for MRD assessment in MM. NGF employs innovative protocols for sample preparation, antibody panel construction, and cell acquisition to address the lack of standardization and inconsistencies associated with conventional flow cytometry [27].

The EuroFlow consortium suggests a two-tube, eight-color combination to identify both surface and intracellular markers, assessing the immunophenotypic characteristics and clonality of neoplastic plasma cells. In the first tube, the main plasma cell (PC) identification markers (CD138, CD38) are combined with CD45, CD19, CD56, CD81, CD117, and CD27. These combinations can distinguish aberrant plasma cells from other cell lineages and normal bone marrow plasma cells. In the second tube, antibodies bound to kappa and lambda cytoplasmic light chains are added, along with CD138, CD38, and the four most common markers (CD19, CD45, CD56, and CD27). The proposed antigenic panel is analyzed in Table 2 [27,28].

The NGF initiative suggests a bulk-lysis procedure using a 0.5% bovine serum albumin lysis solution, which allows acquisition of significantly greater numbers of cells (approximately 10^7^ cells), resulting in a sensitivity advantage compared to conventional flow cytometry. The NGF protocol also involves automatic gating features, reducing bioinformatic labor and turnaround time compared to expert-based conventional MFC [28,29,30]. Expert-controlled data interpretation is one of the main causes of inter-laboratory result discrepancies. This automation improves both the consistency and efficiency of MRD detection, reducing inter-laboratory discrepancies.

While both conventional MFC and NGF rely on immunophenotypic detection of plasma cells, NGF offers several advantages. Conventional MFC, typically employing 4–8 color panels, achieves sensitivities of 10^−4^ to 10^−5^ and is widely available at relatively low cost. However, it suffers from inter-laboratory variability due to lack of standardization in antibody panels, gating strategies, and cell acquisition thresholds. In contrast, NGF, as developed by the EuroFlow Consortium, utilizes standardized 8-color, 2-tube panels and optimized bulk-lysis protocols that allow the acquisition of up to 10^7^ cells, reaching sensitivities of 10^−6^, comparable to next-generation sequencing (NGS) [27]. Moreover, NGF integrates automated gating and data analysis algorithms, reducing operator-dependent variability and enabling harmonization across laboratories [28]. While MFC remains a widely available, cost-effective, and practical tool for diagnosis and monitoring, NGF represents a standardized evolution with superior sensitivity and reproducibility, making it particularly valuable in clinical trials and regulatory contexts. The main distinctions are outlined in Table 1. A visual comparison of MFC, NGF and NGS for MRD assessment is presented in Figure 2, complementing the data summarized in Table 1.

## 4. Current Applications of Multiparameter Flow Cytometry in Multiple Myeloma

### 4.1. Detection of Plasma Cells

MFC provides a fast and widely applicable method for detecting plasma cells in bone marrow, peripheral blood, or any other compartment. The technique primarily relies on evaluating light scatter characteristics and detecting certain immunophenotypic markers. The simultaneous assessment of CD138 and CD38 constitutes the most specific combination for plasma cell identification [13,25].

CD38 is expressed throughout various stages of B-cell maturation but is uniquely upregulated in plasma cells. This high expression level distinguishes plasma cells from other CD38-positive hematopoietic cells, such as monocytes and activated lymphocytes [31,32]. CD38 has also become a therapeutic target with monoclonal antibodies such as daratumumab and isatuximab, which interfere with CD38-based detection. Newer MFC technologies utilize alternative multiepitope and nanobody CD38 antibodies conjugated with fluorochromes; consequently, CD38-based detection is feasible even in patients receiving anti-CD38-based regimens [33].

CD138 (syndecan-1) is primarily expressed on plasma cells and minimally on other hematopoietic cells, representing another highly specific marker for plasma cell identification [15]. However, significant CD138 downregulation has been observed in MM samples not processed within 24 h after collection in heparin-containing tubes, as heparin exposure may lead to CD138 redistribution on the cell surface [11]. Plasma cells expressing low levels of CD138 have been reported, mainly in peripheral blood [34].

Along with CD38 and CD138, MFC uses additional markers such as CD45 and scatter characteristics to distinguish plasma cells from other hematopoietic cells. Plasma cells, being terminally differentiated B cells, typically express some common B-cell markers like CD19 and CD45, although CD20 expression is lost from the plasmablast stage onwards [13,15,25].

Flow cytometry is not only capable of detecting plasma cells but also assessing aberrant immunophenotypes and their clonality, which is a hallmark of malignancy. Neoplastic plasma cells present a distinct immunophenotypic spectrum compared to normal bone marrow plasma cells [27]. Since the first description almost 25 years ago [32], additional studies have verified that neoplastic plasma cells typically present lower expression of [27] CD45, CD19, CD27, and CD81, and elevated expression of CD28, CD33, CD56, CD117, and CD200. It is also worth noting that CD38 expression is weaker in tumor plasma cells compared to their normal plasma cell counterparts.

As mentioned above, immunophenotypic heterogeneity exists due to factors such as sample preparation and processing time. It is important to emphasize that an aberrant immunophenotypic profile cannot rely on one specific marker. Within the plasma cell population, certain markers typically considered aberrant may still be expressed on a subset of normal plasma cells. For example, CD56, while commonly associated with malignant plasma cells, can also be expressed in a small subset of normal or reactive plasma cells. Additionally, although lack of CD19 expression may suggest neoplastic behavior, approximately 30% of normal PCs are also CD19-negative. Therefore, a combination of multiple markers and confirmation of clonal nature is required for highly sensitive and accurate plasma cell detection [27].

For instance, long-lived normal plasma cells tend to downregulate CD19, CD38, and CD45 and upregulate CD56 and CD28, resembling a more malignant immunophenotype. Additionally, samples from MM patients not processed within 24 h present lower CD138 expression [32,34]. It is also worth mentioning that immunophenotypic characteristics differ between bone marrow and circulating tumor plasma cells. Peripheral blood circulating tumor plasma cells (CTPCs) present a more immature and less proliferative immunophenotype than their bone marrow counterparts. They display lower levels of CD38 and CD138, as well as markers that act as stromal adhesion molecules, such as CD56, CD81, and CD117 [35]. It should be noted that some markers, such as CD229 and CD307, are expressed on both normal and aberrant plasma cells, although clonal plasma cells typically exhibit higher expression levels. Similarly, markers such as CD45 and CD117 may show variable expression in both populations, underscoring the importance of interpreting these markers in combination rather than in isolation.

Moreover, the immunophenotype of PCs is dynamic, as malignant PCs are prone to major antigenic shifts upon therapy. Small alterations in immunophenotype may occur because of clonal selection of chemoresistant cells [36]. This phenotypic shift may complicate MRD assessment and emphasize the significance of diagnostic samples in better understanding disease evolution [34]. Immunophenotypic differences between normal and aberrant plasma cells are presented in Table 3. Longitudinal immunophenotyping can reveal phenotypic shifts in aberrant plasma cells, reflecting clonal evolution under therapeutic pressure. Such dynamic monitoring may complicate MRD assessment, as antigenic changes can challenge consistent detection, but it also allows early recognition of resistant subclones. Although not yet standard of care, the integration of serial immunophenotyping represents a promising avenue for personalized treatment approaches, particularly in high-risk patients with recurrent relapses.

### 4.2. Differential Diagnosis

Multiparameter flow cytometry (MFC) serves as a critical tool for differential diagnosis of various hematologic conditions, including MM, complementing morphology and bone marrow biopsy. In the context of suspected plasma cell dyscrasias, MFC is particularly valuable in distinguishing MM from precursor conditions like monoclonal gammopathy of undetermined significance (MGUS) [37]. This differentiation is achieved through quantification of both malignant and normal plasma cell populations within the bone marrow. MGUS is typically characterized by the presence of a mixture of malignant and normal plasma cells, with the latter representing a significant portion. Studies indicate that 98% of MGUS cases exhibit more than 3% normal plasma cells alongside polyclonal cytoplasmic light chain production—features distinct from MM, where malignant plasma cells dominate [37].

Moreover, a study conducted by Paiva et al. identified a subgroup of MM patients with an “MGUS-like” immunophenotypic profile, accounting for approximately 14% of cases [38]. Notably, these patients had more than 5% residual normal plasma cells at diagnosis, marking them as outliers in terms of disease progression and survival. Identification of this subgroup of patients has substantial prognostic implications, as these patients experience more favorable outcomes, including longer progression-free survival and overall survival [38]. In addition to superior treatment outcomes, the “MGUS-like” subgroup has been associated with favorable baseline clinical characteristics, such as lower frequency of anemia, reduced β2-microglobulin levels, lower serum M-protein concentrations, less frequent high-risk cytogenetic abnormalities, and significantly lower rates of immunoparesis [38,39]. From a clinical perspective, recognizing this subgroup may help avoid overtreatment, support less intensive monitoring strategies, and highlight candidates for treatment de-escalation in the context of MRD-adapted approaches. Although not yet part of formal risk models, the MGUS-like phenotype illustrates the potential of flow cytometry to refine prognostic assessment beyond conventional cytogenetics.

In addition to distinguishing MM from precursor conditions like MGUS, MFC is also valuable in differentiating MM from other hematologic malignancies, such as B-cell lymphomas with plasma cell differentiation [15]. One of the most common examples is lymphoplasmacytic lymphoma/Waldenström’s macroglobulinemia, where neoplastic cells produce monoclonal proteins detectable through serum protein electrophoresis and immunofixation. MFC aids in this differential diagnosis by assessing CD19 expression, which is positive in the vast majority of lymphoma-associated plasma cells but negative in over 90% of MM cases. Additionally, CD56, a marker frequently expressed in approximately 75% of MM plasma cells, is much less commonly found in plasma cells associated with non-Hodgkin lymphomas (NHLs), appearing in only about 33% of cases [15,40].

Despite the usefulness of immunophenotyping in MM diagnosis, it should be noted that MFC is recommended only for clonality assessment through kappa and lambda light chain restriction, and not for plasma cell enumeration, according to the International Myeloma Working Group (IMWG) diagnostic criteria for MM [1]. This is because the accuracy of absolute plasma cell counts by MFC can be affected by pre-analytical variables, such as sample hemodilution, processing delays, and variability in acquisition thresholds. Morphological evaluation of bone marrow aspirates remains the gold standard for plasma cell enumeration, whereas MFC provides complementary information by distinguishing clonal from normal plasma cells. In clinical practice, this limitation underscores the importance of integrating MFC results with conventional morphology and histopathology, ensuring that discrepancies in plasma cell percentages are interpreted in the appropriate context.

### 4.3. Response Assessment

Conventional response assessment relies on evaluating monoclonal protein concentrations in serum and urine by electrophoresis and immunofixation, supplemented by free-light chain quantification [4]. Morphological studies of bone marrow aspirate accompany biochemical studies through direct identification of plasma cells by microscopy, with sensitivity around 10^−2^. With current response assessment techniques, approximately 68% of complete response (CR) patients relapse within 2 years [41]. This fact highlights the necessity to renew traditional response criteria and implement more sensitive techniques that surpass the sensitivity limit of 10^−2^. Additionally, serum protein electrophoresis with immunofixation does not exclusively reflect bone marrow compartment occupation due to the half-life of circulating paraprotein [4,19,37].

Flow cytometry using 4–6 colors has proven to be a more sensitive technique that may achieve a sensitivity level of 10^−4^, although still inferior to molecular methods. Recent advancements, particularly NGF with simultaneous assessment of ≥8 markers, may accomplish sensitivity of 10^−6^, which is comparable to NGS [29]. The 2016 International Myeloma Working Group (IMWG) criteria endorsed multiparameter flow cytometry (MFC) for MRD evaluation in CR patients, cementing its role in response assessment and prognostication [4]. NGF has addressed many concerns about standardization and variability between research centers, offering a robust, standardized protocol that includes improved sample preparation, advanced antibody panels, and automated gating features for enhanced sensitivity [4,23,29].

The second tube not only provides information concerning plasma cell clonality but also plays a confirmatory role regarding results exported from the first tube through a second independent measurement. Flores Montero et al. highlighted NGF’s efficacy through comparison with 8-color single tube flow cytometry. A discrepant number of cases was observed, as 25% of patients classified as MRD-negative by conventional 8-color flow cytometry were MRD-positive by NGF. This was largely due to NGF’s ability to evaluate cytoplasmic kappa and lambda light chains, which helped distinguish between monoclonal and polyclonal plasma cells, thus minimizing false positives [28,29,32,42].

Additionally, a cost-effective alternative method was proposed using single-tube, 10-color flow cytometry assay, showing high levels of concordance (98%) with the EuroFlow 8-color, 2-tube assay [43]. However, the two-tube method can acquire 15% more cells due to combined surface and cytoplasmic staining. It should be mentioned that the latter assay has not been standardized, and validation of the method was made on a small patient population, so further investigation is needed [4,43].

Furthermore, extensive research has been conducted to establish MFC and NGF as key instruments in response assessment at the same level of accuracy as molecular methods. In 2019, researchers investigating post-consolidation MRD results from the CASSIOPEIA trial reported excellent levels of concordance between MFC and NGS. In patients achieving CR or greater, there was 94.4% agreement between the two methods [44]. Also, NGS was compared to second-generation MFC showing good levels of agreement, although some discrepancies were attributed to different sensitivity levels between the two techniques. Medina et al. compared NGS and NGF for MRD assessment, also proving excellent levels of agreement. These findings reinforce and verify the establishment of MFC, and especially NGF, as very sensitive and valuable tools for response assessment and patient monitoring [45].

### 4.4. Prognostication

#### 4.4.1. Prognostication Through the Assessment of Minimal Residual Disease

To date, prognosis and risk stratification of MM patients are estimated using the International Stage System (ISS) and the Revised International Stage System (R-ISS). However, additional prognostic factors should be considered, such as circulating plasma cells, extramedullary disease, gene expression profiles, and others, offering great heterogeneity in patient outcomes. High-risk patients often experience inferior survival even when achieving similar responses to therapy as standard-risk patients [4]. Thus, the diversity of different prognostic factors contributes to heterogeneity in predicting patient outcomes. Additionally, these prognostic models using single-step modeling generally rely on pre-treatment factors without integrating depth of response to therapy [46].

The International Myeloma Working Group (IMWG) has emphasized the use of minimal residual disease (MRD) as the preferred biomarker for assessing treatment response. MRD status surpasses traditional criteria, such as complete response (CR), as MRD-negative CR patients have been shown to experience significantly longer survival compared to MRD-positive CR patients. The IMWG strongly recommends using multiparameter flow cytometry (MFC), particularly Next Generation Flow (NGF), to assess MRD in MM patients due to its high sensitivity, specificity, and feasibility [4].

Extensive research [6,9,47] has validated the prognostic advantage of patients who achieve MRD negativity. Achieving MRD negativity identifies a subset of patients with markedly reduced risk of disease progression. Although multiple studies and meta-analyses have demonstrated the prognostic value of MRD, a synthesis of their outcomes underscores the consistency of findings across different methodologies and thresholds. Key trials and analyses are summarized in Table 4. The PETHEMA/GEM2012MENOS65 trial demonstrated that accomplishing MRD negativity before maintenance therapy was associated with an 82% reduction in progression risk and an 88% reduction in death risk [28]. In this group, median PFS was 63 months, whereas in patients achieving only CR, median PFS was 27 months [28]. Similarly, Lahuerta et al. showed that MRD-positive patients in CR had identical outcomes to patients achieving very good partial response (VGPR) and partial response (PR), with PFS between 27 and 29 months [48].

One large meta-analysis conducted by Munshi et al. analyzed data from 44 studies and 4297 patients and confirmed that MRD negativity is associated with remarkably improved survival outcomes, regardless of disease setting (newly diagnosed or relapsed/refractory disease), thresholds used for MRD, cytogenetic risk, and method used for MRD assessment [6]. Compared to MRD-positive patients, achievement of MRD negativity was associated with significantly improved PFS (HR, 0.33; 95% CI, 0.29–0.37; *p* < 0.001) and OS (HR, 0.45; 95% CI, 0.39–0.51; *p* < 0.001). Multivariate analysis further demonstrated that achievement of MRD negativity is an independent prognostic factor, regardless of age, disease stage, cytogenetics, or transplant eligibility [6].

Risk is a dynamic factor, since patients with a non-favorable prognosis may shift into a better prognosis through accomplishment of MRD negativity. Thus, patients with initially poor prognoses can achieve outcomes comparable to lower-risk patients if MRD negativity is reached. This dynamic nature of risk suggests that MRD negativity should be pursued across different patient subgroups, including older and transplant-ineligible individuals, to ameliorate patient outcomes [28,48,49].

The most recent and largest meta-analysis (EVIDENCE meta-analysis) conducted by Landgren et al. examined data from over 4900 patients across multiple clinical trials, confirming that MRD negativity at 12 months is strongly correlated with improved progression-free survival (PFS) and overall survival (OS), with global odds ratios of 4.02 for PFS in newly diagnosed patients and 7.67 in the relapsed/refractory setting. These findings suggest that MRD negativity is a robust prognostic marker that can accelerate drug approvals by serving as an early clinical endpoint, further supporting its utility in guiding therapeutic decisions [50]. Similarly, the MASTER trial demonstrated that 81% of patients reached MRD negativity with quadruplet induction therapy, and those who achieved a sustained MRD-negative, treatment-free state (termed MRD-SURE) had excellent long-term outcomes, including high 3-year OS and PFS rates, irrespective of cytogenetic risk. This evidence highlights MRD negativity as an essential marker for guiding treatment and risk stratification in both standard and high-risk MM patients [51].

Thus, application of MFC and NGF in MM patients for MRD evaluation provides a robust tool for improving risk stratification, assessing therapeutic efficacy, and identifying those at higher risk of short-term progression. MRD negativity represents a critical goal for achieving improved patient outcomes, offering insights that surpass the limitations of conventional response criteria [42,49].

**Table 4:** Key studies evaluating the prognostic impact of MRD negativity on outcomes in multiple myeloma.

Study/Trial	Method of MRD Assessment	MRD Threshold	Key Outcomes (MRD− vs. MRD+)
Paiva et al., [38]	4-color MFC	10^−4^	MRD– patients had significantly longer PFS and OS
Rawstron et al., (meta-analysis) [52]	MFC (various panels)	10^−4^–10^−5^	MRD negativity consistently associated with improved PFS and OS across trials
Munshi et al., (meta-analysis) [9]	MFC/NGF/NGS	10^−4^–10^−6^	MRD negativity associated with 50% reduction in risk of progression/death
Avet-Loiseau et al., [44]	MFC/NGS	10^−5^	MRD negativity strongly correlated with longer PFS after consolidation
Paiva et al., PETHEMA/GEM2012MENOS65 [28]	NGF (EuroFlow)	10^−6^	MRD– patients had 5-year PFS > 80%, MRD+ significantly worse outcomes
Costa et al., [51]	NGF (EuroFlow)	10^−5^–10^−6^	Sustained MRD negativity (MRD-SURE) associated with excellent 3-year OS and PFS

#### 4.4.2. Prognostication Through Immunophenotypic Expression Characteristics

Persistence of residual tumor burden after treatment correlates with worse outcomes. However, there is considerable heterogeneity in survival outcomes among MRD-positive patients. Recently, with the ability to detect extremely low levels of residual disease, there is also a growing need to stratify MRD-positive patients. Unlike other MRD assessment techniques, flow cytometry offers additional insights into tumor biological behavior through immunophenotypic analysis, providing valuable prognostic information [15,53].

One of the most commonly studied markers on the surface of malignant plasma cells is CD56, which is expressed in approximately 70–80% of multiple myeloma (MM) cases. Studies have shown that the absence of CD56 correlates with poor prognosis, including elevated levels of lactate dehydrogenase (LDH), increased β2-microglobulin, higher International Staging System (ISS) stage, and greater bone marrow infiltration. Additionally, CD56 expression showed a more mature degree of plasma cell differentiation. However, the effect on overall survival in CD56-negative patients remains a controversial issue of extensive research [54].

The expression of CD200 has also garnered significant attention. CD200 is present in about 70–75% of aberrant plasma cells in MM. CD200-positive patients tend to have significantly shorter OS compared to their CD200-negative counterparts (*p* = 0.009) [55]. The adverse effect of CD200 expression is believed to be immunomodulatory, involving dysregulation of the CD4/CD8 T-cell ratio, particularly through an increase in CD8 T-cells. Some studies suggest that dynamic changes in CD200 expression during treatment may have prognostic value, with many patients shifting to CD200 negativity, which is associated with improved survival outcomes. However, dynamic changes in CD200 expression may also be of prognostic value. Most patients shift to CD200 negativity through the course of treatment. Those patients had favorable survival outcomes compared to others. Consequently, baseline detection of CD200 through flow cytometry and monitoring its expression throughout the disease course may extract prognostic information. However, to date, CD200 is not one of the commonly used markers suggested by the EuroFlow consortium [55].

Sahara et al. demonstrated that MM patients expressing CD33 exhibited higher levels of β2-microglobulin and LDH, as well as higher incidence of anemia and thrombocytopenia. Their study found that CD33-positive patients had significantly shorter 3-year survival compared to CD33-negative patients (*p* = 0.042). CD33 expression was linked to drug resistance, as it increased in some patients following treatment [56]. Another key marker, CD28, is expressed in the majority of aberrant plasma cells and becomes more prevalent as the disease progresses, particularly in advanced stages such as extramedullary disease, relapse, or secondary plasma cell leukemia [13].

Recently, Zohar et al. investigated the prognostic impact of CD24. CD24 is a glycoprotein found in the membrane surface that is thought to stimulate cell adhesion to the bone marrow extracellular matrix. Its role has also been studied in solid tumors, where it has been correlated with metastatic disease formation and poor prognosis. It acts as a differentiation marker on B-cell progenitors, and its expression decreases once lymphocytes enter the germinal center and undergo terminal differentiation. In MM, CD24 expression presents a dynamic pattern, with CD24 levels being elevated in progressive stages of the disease. However, contrary to solid malignancy behavior, in MM higher CD24 expression is correlated with greater survival rates and overall better prognosis, as CD24-positive plasma cells present less tumorigenic behavior than CD24-negative plasma cells. Furthermore, in a series of 124 patients with newly diagnosed MM or primary amyloidosis, it has been shown that patients with CD24 expression (with a cut-off level of 5% on PCs at diagnosis) have a median PFS of 36.2 months, compared to 22.8 months for patients with lower expression levels (*p* = 0.002) [57].

Moreover, Chen et al. explored the prognostic impact of CD81 and CD117 expression. They found that CD117+ patients had longer PFS and overall survival (OS), whereas CD81+ patients had shorter PFS and OS. Based on CD81 and CD117 expression, patients were categorized into three groups: CD117+/CD81− (good prognosis), CD117−/CD81+ (poor prognosis), and a mixed group (intermediate prognosis) [35].

Arana et al. studied the prognostic significance of various immunophenotypic markers in 1265 patients enrolled in the GEM/PETHEMA studies. They found that the combination of CD19+, CD27−, CD38low, CD45+, CD81+, CD117−, and CD138low was associated with worse outcomes. Multivariate analysis revealed that the CD38low, CD117−, and CD81+ phenotype had an independent adverse impact on survival, with significantly lower PFS (median 22 vs. 35 months) and OS (median 43 vs. 76 months). The study also noted that a phenotypic combination of CD138high, CD38high, and CD19- was associated with superior survival outcomes. Furthermore, the study observed antigen expression shifts during MRD assessment, particularly with CD81. These shifts were detected in approximately 17% of patients, primarily involving CD81, and did not affect overall MRD detection [53].

Another critical application of MFC is the ability to provide real-time information on other parameters beyond MRD, offering insights into the biological behavior of the malignancy. Paiva et al. analyzed 40 elderly MM patients enrolled in the GEM2010MAS65 study to characterize the immunophenotypic and genetic features of chemoresistant MRD clones. Using 8-color flow cytometry, they identified significant upregulation of integrins such as CD11a, CD11c, CD29, CD44, CD49d, CD49e, CD54, CD138, CXCR4, and HLA-DR in these resistant cells [58]. These phenotypic differences are considered to result from therapy-induced phenotypic selection between diagnosis and MRD stage, showing greater expression of integrins and adhesion molecules, giving these resistant cells a survival advantage [58,59]. In summary, immunophenotypic profiling through flow cytometry has emerged as a powerful tool in understanding the prognostic landscape of multiple myeloma, offering crucial insights into patient outcomes and resistance mechanisms.

Although cytogenetic abnormalities detected by FISH remain the standard for risk stratification in multiple myeloma, it is important to recognize the potential of flow cytometry-derived immunophenotypic markers as prognostic tools. Specific antigen expression patterns, such as CD28 positivity (associated with advanced disease and extramedullary spread) and CD56 negativity (linked to extramedullary plasmacytoma and poor prognosis), provide valuable risk information at lower cost and with greater accessibility than FISH [54,56]. Similarly, CD200, CD117, and CD81 have shown independent prognostic significance in large cohorts [35,53,55]. Despite this evidence, flow cytometry-based risk stratification has not yet been incorporated into standard models, largely due to the need for prospective validation and harmonization of methodologies.

## 5. Circulating Tumor Plasma Cells (CTPCs) and Peripheral Blood Assessment

Despite bone marrow being the most frequently involved tissue in plasma cell neoplasms, several studies report involvement of circulating tumor plasma cells in most patients. Plasma cell identification in peripheral blood depends on the subtype of plasma cell neoplasm, biological behavior, and sensitivity of the technique used. With conventional microscopy, due to its limited sensitivity and small number of assessed cells, CTPCs are identified only in a subset of patients with significant disease dissemination and aggressive disease behavior [60,61]. Currently, using more sensitive techniques such as MFC and NGF, CTPCs are found in the majority of treatment-naïve patients, and higher CTPC levels are associated with worse clinical outcomes [62].

Sanoja Flores et al. [60], utilizing NGF, showed that CTPCs may be found in 70% of patients with plasma cell neoplasms. The frequency of CTPC identification gradually increased from patients with solitary plasmacytoma (18%) to MGUS (59%) to smoldering multiple myeloma (SMM) and MM (100%). Additionally, since every CTPC-positive case is also MRD-positive, enumeration of CTPCs may indicate a surrogate marker of persistent disease [60]. Thus, use of CTPCs as a biomarker for disease evaluation has attracted attention due to the minimally invasive nature of liquid biopsies, precise enumeration of CTPCs, independence from hemodilution and the patchy nature of MM, and the association between CTPC numbers and disease aggressiveness [60,62,63].

Extensive research has been conducted using flow cytometry studies to support the correlation between CTPC numbers and disease dissemination and to establish CTPC quantification as a disease severity biomarker. Key studies that have been conducted to support the prognostic impact of CTPCs are summarized in Table 5. Sanoja Flores et al., using ROC curve analysis and a cut-off of 0.058 CTPCs/μL, identified MGUS cases with increased risk of progression to symptomatic MM and a small subset of MM cases with more benign clinical behavior (MGUS-like phenotype) that display significantly longer survival and time-to-progression based on their CTPC absolute numbers [60]. Multiple studies have supported the notion that high levels of CTPCs strongly correlate with worse outcomes in newly diagnosed multiple myeloma (NDMM). Multivariable analysis showed that patients with advanced ISS stage (ISS II and III), increased levels of LDH and β2-microglobulin, and high-risk cytogenetics present increased levels of CTPCs [62,63,64,65].

Kostopoulos et al. analyzed 525 matched bone marrow and peripheral blood samples from patients with NDMM using NGF. After median follow-up of 42 months, investigators used a cut-off of 0.02% of CTPCs for risk stratification. NDMM patients with CTPCs > 0.02% showed a 2.2-fold higher risk of progression compared to patients with CTPCs < 0.02% (36 vs. 60 months), which was independent of baseline characteristics. Immune composition analysis showed that patients with increased CTPCs presented a plasma cell leukemia-like profile in terms of cell migration, proliferation, and DNA damage control. These patients presented increased numbers of CD8+ T-cells, NK cells, tumor-associated macrophages, and an elevated ratio of memory to naïve B cells, indicating more cytotoxic behavior [66].

Garcés et al., using NGF, also proved the prognostic significance of CTPCs in a total of 374 transplant-eligible patients with NDMM [67]. Five-year rates of PFS progressively deteriorated in cases with increasingly higher logarithmic percentages of CTPCs, from >0.0001% to >1%. A cut-off of 0.01% was used for risk stratification, showing that patients with CTPCs > 0.01% had inferior PFS and OS than patients with CTPCs < 0.01%. That study aimed to incorporate CTPC count with other most used prognostic factors (ISS stage, lactate dehydrogenase levels, and cytogenetics) to improve risk stratification. With median follow-up of 5 years, 90% of patients with undetectable CTPCs after receiving fixed-duration maintenance therapy remain progression-free. Another study conducted by Sathya et al., using a baseline cut-off of 0.197%, showed that there was significant association between CTPC percentage at baseline and depth of response (*p* = 0.008), as patients with baseline CTPCs < 0.197% tend to have deeper responses (VGPR and above) post-induction [67].

Primary plasma cell leukemia (pPCL) is the most aggressive form of plasma cell dyscrasias. It was formerly characterized by ≥20% plasma cells in peripheral blood until 2021, when diagnostic criteria were revised and the threshold was decreased to ≥5%. Jelinek et al. used 8-color flow cytometry to give prominence to the dismal prognosis of patients with increased CTPCs, assessing a total of 590 transplant-eligible and transplant-ineligible patients [68]. They used a clinically relevant cutoff of 2%, dividing patients into two groups: one being patients with <2% CTPCs and the other being patients with CTPCs from 2 to 20%. Patients with >2% CTPCs were compared to a pPCL patient cohort. Overall survival rates were identical, and the difference was not statistically significant (13 vs. 14.6 months, *p* = 0.908). Thus, using flow cytometry, they established a threshold of 2% to identify extremely high-risk patients with pPCL-like clinical behavior and similar outcomes [61,68,69]. Clinically, the detection of high levels of CTPCs has direct implications for both diagnosis and risk assessment. According to IMWG criteria, a peripheral blood plasma cell count ≥5% defines primary plasma cell leukemia (pPCL), an entity with aggressive clinical course and poor prognosis. Furthermore, even below this diagnostic threshold, elevated CTPC levels (e.g., ≥0.01–0.1% by MFC/NGF) have consistently been associated with adverse outcomes in newly diagnosed MM. In practice, this means that quantifying CTPCs at diagnosis may help identify a subgroup of high-risk MM patients who, while not fulfilling criteria for pPCL, still require closer clinical monitoring and may benefit from enrollment in clinical trials or more intensive therapeutic strategies.

In summary, detection of CTPCs using flow cytometry may provide valuable insights into management and risk stratification of MM patients. CTPCs serve as a surrogate biomarker of great significance to assess disease burden and dissemination, as higher counts of CTPCs are an independent prognostic factor associated with dismal outcomes. Evaluation of peripheral blood through flow cytometry provides a minimally invasive approach, not affected by patchy disease distribution and hemodilution, to monitor treatment response and therefore prognosis of MM patients. Extensive research has been and is ongoing to validate the role of flow cytometry studies in peripheral blood and to establish this technique as a first-line component of patient monitoring [64,67].

## 6. Immune Composition Assessment Using Flow Cytometry

Multiple myeloma is characterized by proliferation of neoplastic plasma cells in the bone marrow compartment, altering the bone marrow microenvironment and immune cell composition. Interactions between cellular components and dysregulation of the immune profile create an immunosuppressive state, rendering the bone marrow susceptible to tumor cell proliferation and drug resistance [70]. Flow cytometry has been applied to assess immune cell profiles at different stages of the disease and associate immune composition with patient outcomes. The NGF protocol allows evaluation of the whole bone marrow compartment and monitoring of cellular composition alterations that may arise during relapse [71].

Multiple myeloma patients achieving long-term disease control present a considerably different immune profile compared to patients with symptomatic multiple myeloma and healthy patients. Pessoa de Magalhães et al. [72] used flow cytometry to compare immune profiles of multiple myeloma patients in long-term remission with those who had symptomatic MM. They found that patients in long-term remission had an expanded population of CD8+ T-cells and natural killer (NK) cells, along with a lower CD4/CD8 ratio, suggesting a more cytotoxic immune environment. This immune profile was associated with better disease control and outcomes, highlighting the role of immune surveillance in long-term disease remission [72].

Paiva et al. [73] used eight-color flow cytometry to investigate whether immune profile at the time of MRD assessment was of prognostic significance. It categorizes patients into three immune profile groups based on cell counts of B-cell precursors, mature B-cells, and erythroblasts. The study reported that patients with more favorable immune composition—characterized by higher counts of mature naïve and memory B-cells—had significantly better overall survival and progression-free survival, even among MRD-positive patients. These data highlight the prognostic value of immune profiling alongside MRD monitoring [73].

Luoma et al. categorized patients into two groups based on their response to therapy and investigated their respective immune profiles. Using flow cytometry, they found that patients responding well to treatment had increased cytotoxic T-cell and NK-cell populations with higher expression of cytotoxicity markers (such as CD45RA and CD57). Patients who responded poorly showed higher numbers of CD4+ T cells and memory cells, indicating a less effective immune response. This study suggests that immune profiling can provide insights into mechanisms of response and resistance to immunomodulatory treatments like lenalidomide [70].

Krzywdzinska et al., using the EuroFlow panel, assessed bone marrow microenvironment immune composition alterations in patients at biochemical relapse. The study reported a reduction in B-cell precursors and an increase in memory B cells during disease progression, along with a slight rise in T-cell and NK-cell numbers. These findings suggest that patients at relapse exhibit a shift toward a more memory-dominant immune profile, which could potentially impact disease recurrence and treatment resistance. This profiling offers insights into the immune system’s involvement in MM progression [71].

In addition to quantifying immune cell composition, MFC provides prognostic immune signatures that can inform patient outcomes. The integration of MFC with single-cell technologies—such as single-cell RNA sequencing or high-dimensional cytometry (CyTOF)—holds great promise for deeper dissection of immune system–tumor interactions. Such multimodal approaches can uncover novel mechanisms of immune escape, identify biomarkers predictive of response to immunotherapies (e.g., monoclonal antibodies, bispecific antibodies, CAR-T cells), and ultimately support the optimization of immunotherapy strategies.

## 7. Other Perspectives

The establishment of MFC for monitoring MM patients is an achievement of recent years, mainly aiming for MRD detection, predominantly for prognostication purposes. The purpose of MRD evaluation in MM is to extract information regarding disease status on which clinical decisions should be based. Therefore, it is necessary that MRD assessment should provide reproducible, reliable, prognostically relevant, and quick answers to face clinical uncertainties and dilemmas, differentiating patients who are likely to benefit from certain interventions. Since multi-drug treatment regimens come with increased toxicity, especially in older, frail patients, evaluation of MRD should provide guidance concerning therapy regimens, intensity, and duration based on MRD status. For instance, MRD positivity after treatment raises the question of whether it is necessary to change the treatment regimen or address treatment intensification to improve response depth. MRD negativity after therapy may be an indication for treatment de-escalation or even discontinuation of maintenance therapy, especially in patients with many comorbidities [19,22]. However, to date, MFC is not routinely used as a tool to provide treatment decision guidance. The impact of MRD assessment in clinical decision-making needs to be determined, and extensive research is ongoing to validate its significance. MFC and especially NGF, as mentioned above, provide a highly applicable and available method of therapy response evaluation that can be assessed at any point of therapy without the need for a baseline sample. However, even though the prognostic significance of MRD is validated, extensive debate is ongoing about its predictive value and whether it should be used for guidance in clinical decision-making. Further studies are required to establish the predictive value of MRD assessment.

The incorporation of MRD into treatment decision-making represents one of the most promising applications of flow cytometry in MM. Several recent clinical trials have adopted MRD-adapted strategies, where therapy is either escalated or de-escalated depending on MRD status. For example, the MASTER trial used sustained MRD negativity to guide treatment cessation, allowing many patients to discontinue therapy while maintaining excellent long-term outcomes [51]. Similarly, in the PETHEMA/ GEM2012MENOS65 trial, MRD-positive patients after consolidation had significantly shorter PFS and OS, supporting the rationale for therapy intensification in this subgroup [28]. Flow cytometry, especially NGF, is ideally suited for such adaptive approaches due to its wide applicability, rapid turnaround time, and lack of requirement for a baseline diagnostic sample [27].

In clinical practice, MRD-adapted strategies could include prolonging or intensifying therapy in MRD-positive patients, while considering treatment de-escalation or discontinuation in sustained MRD-negative patients. Although not yet standard of care, this paradigm is increasingly supported by clinical trial evidence and regulatory discussions. Selected examples of MRD-adapted clinical trials are summarized in Table 6. These results underscore the potential of MRD assessment by NGF/MFC not only as a prognostic tool but also as a guide for individualized therapy. Wider adoption will require consensus on thresholds, timing of assessment, and prospective validation of MRD-driven algorithms.

A key message emerging from recent evidence is that multiparameter flow cytometry may serve not only for MRD detection but also as a powerful, low-cost prognostic tool. Integrating immunophenotypic markers into risk stratification models, either in conjunction with or as a complement to FISH-based cytogenetics, represents an important avenue for future research. We advocate for further prospective, multicenter studies to validate flow cytometry-derived prognostic markers and to establish their role in risk-adapted treatment decision-making (R2C2).

It should be underlined that multiple myeloma is a patchy disease with also the potential presence of extramedullary sites of active disease. Extramedullary disease may be present in approximately 10% of patients at diagnosis and is associated with poor prognosis. Given the spatial heterogeneity of MM and the fact that bone marrow MRD is assessed from a specific point of aspiration, it is crucial that a more systemic approach is mandatory. Paiva et al. demonstrated that even in NGF MRD-negative patients, early relapses still occur, attributed to the presence of extramedullary disease and circulating tumor cells [8,37,48]. Imaging, with PET-CT being the greatest representative, plays a complementary role in bone marrow MRD [74]. Rasche et al. showed that patients who were both flow-MRD and PET/CT-MRD negative had better prognosis, with the best PFS outcome, as compared to flow-MRD negative but PET/CT positive or vice versa [42,75,76]. MRI is an imaging technique with great value at diagnosis, according to the IMWG criteria [4]. However, focal lesions may take time to disappear, even in patients responding to therapy, thus making MRI an inferior tool for response assessment compared to PET-CT. The answer to distinguishing active disease and bone remodeling through MRI was given through functional Diffusion Weighted Imaging (DWI). MRI with DWI assays presents comparable results to PET/CT when addressing response to therapy through imaging [8,77].

In this context, complementary MRD techniques should also be considered. For example, the combined use of NGF and NGS in bone marrow aspirates may provide both rapid, widely applicable detection (NGF) and ultra-sensitive molecular assessment (NGS). Similarly, integration of bone marrow NGF with mass spectrometry of monoclonal proteins may further enhance sensitivity and allow monitoring through peripheral blood. Such hybrid approaches provide a more comprehensive and systematic strategy for MRD detection, increasing sensitivity thresholds and potentially offering particular benefit in ultra-high-risk patients where conventional single-method assessment may be insufficient.

Hemodilution is a main technical issue that may corrupt MRD analysis of bone marrow, giving at times false negative MRD results, even if enough cells are obtained. Thus, it is crucial to evaluate the quality of the aspirated sample through assessing cellularity and analyzing additional cell populations with a high degree of partition between bone marrow and peripheral blood. It is worth mentioning that MFC may provide an overall assessment of the bone marrow compartment and sample quality check, identifying possible sample contamination and hemodilution. These populations include nucleated red blood cells (cells negative for all antigens commonly tested in myeloma MRD tube), mast cells (exceptionally bright CD117 with somewhat increased side scatter), early maturing B cells (hematogones) (CD19-positive cells with bright CD81 and CD38 without cytoplasmic light chain expression with relatively dim CD45), and early myeloid and erythroid precursors (CD117-positive, CD27-negative cells). Evaluation of these additional populations was automated by the EuroFlow assay and can also be easily performed manually outside of the platform [9,26]. Hemodiluted samples are inadequate for MRD detection; therefore, qualitative assessment of bone marrow aspirate is of great significance before drawing further conclusions [10].

## 8. Future Directions

**Figure 3:**
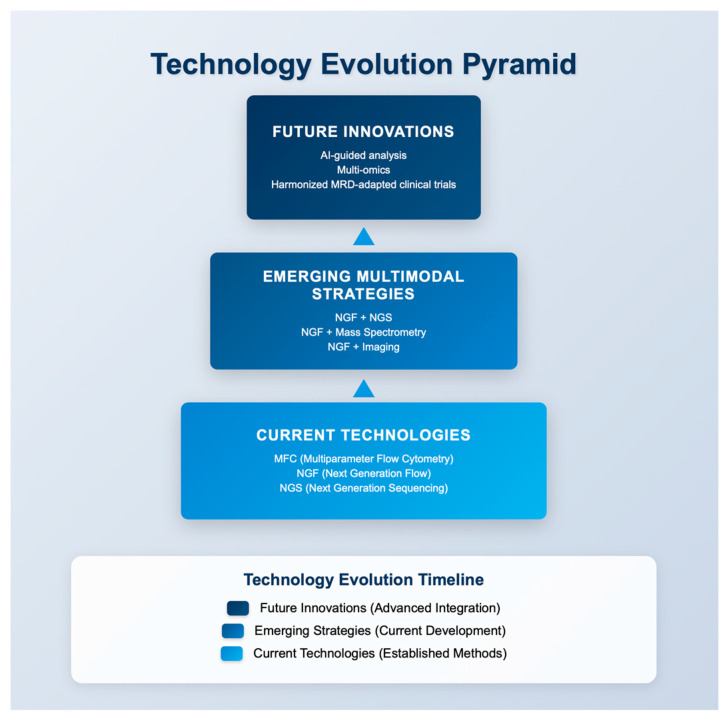
Technology evolution pyramid illustrating current MRD techniques, emerging multimodal strategies, and future innovations. Abbreviations: MFC = multiparameter flow cytometry; NGF = next generation flow; NGS = next generation sequencing; MRD = measurable residual disease; AI = artificial intelligence.

Looking ahead, several emerging technologies are expected to further enhance the role of flow cytometry in multiple myeloma. First, AI-guided analysis and machine learning algorithms can automate gating procedures, minimize operator variability, and increase the sensitivity of assays, thereby enhancing the reproducibility and scalability of MFC. Second, the integration of multi-omics approaches—combining immunophenotyping with genomics, transcriptomics, proteomics, and metabolomics—will deepen insights into clonal evolution and immune system–tumor interactions, potentially unraveling novel biomarkers and therapeutic targets. Third, integration of flow cytometry into clinical trial design is crucial. NGF-based MRD assessment is already being implemented in major clinical trials (e.g., MASTER, GEM2012, MRD2STOP) to determine whether treatment adaptation based on MRD status improves outcomes. However, further large-scale studies are required to validate the predictive value of MRD and to establish its role as a decision-making biomarker for therapy escalation, de-escalation, or discontinuation. Finally, global harmonization of MRD assessment remains a priority. Adoption of standardized EuroFlow protocols and updated IMWG/EMN recommendations, along with international harmonization initiatives, will be essential to ensure reproducibility and comparability of MRD data across centers and healthcare systems. Taken together, these advances suggest that the coming decade will likely see MRD evolve from a powerful prognostic biomarker into a central predictive tool guiding personalized therapy, supported by advanced analytics, multi-omics integration, and globally harmonized methodologies. An overview of emerging directions in MRD assessment, including hybrid methodologies, AI-guided analysis and integration with clinical trials is illustrated in Figure 3.

A critical challenge for the field is the global harmonization of MRD assessment protocols. While EuroFlow NGF has provided a gold standard for sensitivity and reproducibility, widespread implementation outside specialized centers is limited by cost, training requirements, and access to standardized reagents. Ongoing efforts by international groups—including the IMWG, EMN, and the EuroFlow Consortium—are working toward consensus guidelines to reduce inter-laboratory variability and establish MRD as a universally reliable endpoint in clinical trials and practice. Achieving this goal will be essential for MRD-guided treatment strategies to become broadly applicable across healthcare systems. The evolution of MRD assessment technologies, from current approaches to emerging multimodal strategies and future innovations, is illustrated in Figure 3.

## 9. Conclusions

In previous years, immunophenotyping with the use of MFC has not been routinely used for MM patients, unlike other hematological malignancies where MFC had wide acceptance. Currently, consensus exists about the usefulness of MFC, especially in the following disciplines in the MM field: first, differential diagnosis of plasma cell disorders and other hematological malignancies; second, evaluation of response depth and MRD monitoring; and third, the prognostic stratification of MM patients. NGF can be regarded as an evolution of conventional MFC, offering improved sensitivity, standardization, and reproducibility. MFC remains an important and cost-effective tool for widespread clinical practice, whereas NGF provides the methodological rigor needed for clinical trials and regulatory purposes, where the harmonization of MRD assessment is critical. Furthermore, the application of MFC to peripheral blood samples offers a minimally invasive approach to assessing disease dissemination and prognosis, facilitating frequent monitoring throughout treatment. Future strategies will likely rely on multimodal MRD assessment, combining NGF/MFC with molecular assays or imaging. These hybrid approaches may overcome the limitations of individual techniques and provide ultra-sensitive monitoring, particularly critical for patients with high-risk disease. Consequently, it is of great importance that MFC should be incorporated in routine monitoring of MM patients in day-to-day clinical practice, complementing conventional methods of disease follow-up. Continued research is essential to further expand the capabilities of MFC, with the aim of improving patient care and outcomes in MM.

## Figures and Tables

**Table 1:** Comparison of Bone Marrow MRD assessment techniques.

	MFC	NGF	NGS
Applicability	Nearly 100%	Nearly 100%	Around 90%
Availability	Wide	Wide	Limited
Cost	Cost-effective	Cost-Effective	High
Sensitivity	10^−4^–10^−5^	10^−6^	10^−6^
Complexity	Rapid turnaround time	Rapid turnaround time	Labor intensive, requires intense bioinformatic infrastructure
Standardization	Not standardized	Standardized by the EuroFlow Consortium	Standardized
Sample processing	Fresh samples within 24 h	Fresh samples within 24 h	Stored samples can be assessed
Need for baseline sample	No	No	Yes
Clinical utility	Diagnosis, clonality, MRD (limited sensitivity)	MRD monitoring in trials and practice; regulatory use	MRD monitoring, high sensitivity; increasingly used in clinical trials

**Table 2:** Antigens conjugated with fluorochromes proposed by the EuroFlow NGF initiative.

Tube 1 (Surface Antigens)	Tube 2 (Surface and Cytoplasmic Antigens)
CD138-BV421	CD138-BV421
CD38-FITC	CD38-FITC
CD19-PEC7	CD19-PEC7
CD45-PERCP	CD45-PERCP
CD56-PE	CD56-PE
CD27-BV510	CD27-BV510
CD81-APCC750	Lamda-APCC750
CD117-APC	Kappa-APC

**Table 3:** Immunophenotypic differences between normal and aberrant plasma cells.

Marker	Normal Plasma Cells	Aberrant (Clonal) Plasma Cells	Notes
CD19	Positive	Typically negative	Helps distinguish normal vs. clonal PCs
CD45	Positive (variable intensity)	Often negative or dim	Loss associated with clonality
CD56	Negative	Frequently positive	Aberrant expression in ~70% of MM cases
CD200	Usually negative	Frequently positive	Aberrant expression associated with poor prognosis
CD117	Variable	Variable (positive in ~30% of MM cases)	May have prognostic significance
CD81	Strongly positive	Reduced or absent	Loss often indicates clonality
CD229	Positive	Positive (generally higher expression)	Intensity difference aids distinction
CD307 (SLAMF7)	Positive	Positive (generally higher expression)	Target of elotuzumab; higher in clonal PCs

**Table 5:** Summary of key studies evaluating Circulating Tumor Plasma Cells in multiple myeloma.

Study/Year	Method of Detection	CTPC Cut-Off	Patients (n)	Median Follow-Up	Key Outcomes (PFS/OS)
Sanoja-Flores et al., [60]	NGF	0.058 CTPC/μL	264	24 months	CTPC- showed prolonged PFS and OS
Kostopoulos et al., [66]	NGF	0.02%	525	42 months	CTPCs > 0.02% showed higher risk of progression, independent of baseline risk (36 vs. 60 months)
Garces et al., [67]	NGF	0.01%	374	5 years	Progressive deterioration in PFS with higher CTPCs levels, inferior PFS, OS
Sathya et al., [64]	MFC	0.197%	66	Post-induction	Baseline CTPCs < 0.197% results in deeper responses (≥VGPR)
Jelinek et al., [68]	MFC	2%	590	49.1 months, 37.3 months	Patients with CTPCs > 2% showed similar outcomes to pPCL

**Table 6:** Examples of Clinical Trials Incorporating MRD-Adapted Strategies in Multiple Myeloma.

Trials	Method of MRD Assessment	MRD Threshold	Adaptive Strategy	Key Findings
MASTER [51]	NGF (EuroFlow)	10^−5^ to 10^−6^	Therapy cessation after sustained MRD negativity	81% achieved MRD negativity; patients with sustained MRD-negative status (MRD-SURE) had excellent 3-year OS and PFS irrespective of cytogenetic risk
PETHEMA/GEM2012MENOS65 [28]	NGF	10^−5^ to 10^−6^	Therapy intensification in MRD-positive patients	MRD negativity before maintenance reduced risk of progression by 82% and death by 88%
CASSIOPEIA [44]	MFC and NGS	10^−5^	Post-consolidation MRD assessment	High concordance between MFC and NGS; MRD negativity strongly correlated with improved PFS
EMN02/HO95 [30]	NGF	10^−5^	MRD assessment after consolidation and maintenance	MRD negativity associated with prolonged survival, independent of ISS stage and cytogenetics

## Data Availability

Our study is a review of the current international literature, which is analyzed in the reference section. No new data were created or analyzed in this study. Therefore, data sharing is not applicable to this article.

## Abbreviations

The following abbreviations are used in this manuscript:

MFC	Multiparameter Flow Cytometry
NGF	Next-Generation Flow
CTPC	Circulating Tumor Plasma Cell
MRD	Measurable Residual Disease
